# Residual dizziness after BPPV management: exploring pathophysiology and treatment beyond canalith repositioning maneuvers

**DOI:** 10.3389/fneur.2024.1382196

**Published:** 2024-05-24

**Authors:** O. Nuri Özgirgin, Herman Kingma, Leonardo Manzari, Michel Lacour

**Affiliations:** ^1^Bayındır Sogutozu Hospital, Ankara, Türkiye; ^2^Faculty of Medicine, Aalborg University, Aalborg, Denmark; ^3^Maastricht University Medical Center, Maastricht, Limburg, Netherlands; ^4^Vestibology Science, MSA ENT Academy Center, Cassino, Lazio, Italy; ^5^Aix-Marseille Université, Neurosciences Department, Marseille, France

**Keywords:** residual dizziness, benign paroxysmal positional vertigo, vestibular compensation, holistic, pathophysiology

## Abstract

Despite the high success rate of canalith repositioning maneuvers (CRMs) in the treatment of benign paroxysmal positional vertigo (BPPV), a growing number of patients report residual dizziness symptoms that may last for a significant time. Although the majority of BPPV cases can be explained by canalolithiasis, the etiology is complex. Consideration of the individual patient’s history and underlying pathophysiology of BPPV may offer the potential for treatment approaches supplementary to CRMs, as well as a promising alternative for patients in whom CRMs are contraindicated. This article provides a summary of the possible underlying causes of BPPV and residual dizziness, along with suggestions for potential management options that may be considered to relieve the burden of residual symptoms.

## Introduction

Benign paroxysmal positional vertigo (BPPV) is the most common cause of peripheral vestibular vertigo (1). It is characterized by short, repeated episodes of intense vertigo and/or positional nystagmus triggered by specific head position changes relative to the gravity vector, and often accompanied by nausea and vomiting (2). BPPV is more common in women and has an overall prevalence of 2.4% in the general adult population that increases with age (2).

While approximately 50–70% of BPPV cases are primary (idiopathic), the remaining cases are often associated with an underlying pathology, such as head trauma, acute unilateral vestibular pathology (3), labyrinthitis, or Menière’s disease (4). BPPV accounts for 24.1% of all hospital visits due to dizziness/vertigo (5). Recurrences of BPPV are frequent with an annual recurrence rate of 15–20% (6).

## Pathophysiology of BPPV

The five major vestibular sensory organs in the inner ear, the utricle, the saccule, and the lateral, superior and posterior semicircular canals, contain mechanoreceptors (hair cells/stereocilia) that are deflected by accelerations and thereby signal head motion and head tilt via the vestibular nerve to the vestibular nuclei and cerebellum (7). This coordinates the vestibulo-ocular reflex (VOR) and the vestibulospinal reflex (VSR) to maintain orientation and stabilization of the eyes and body that are required for optimal visual acuity during head motion, balance, spatial orientation, navigation and several autonomic functions, as well as cognition. The otolith organs, the utricle, and the saccule, contain hair cells positioned underneath the extrastriolar regions that are covered with heavy otoconia that specifically detect head tilt (gravity) and translational accelerations. The semi-circular canals contain hair cells located in the cupula and by deflection of the cupula specifically detect rotational accelerations of the head.

Canalithiasis has been gradually accepted as the cause of most cases of BPPV (8), a process in which otoconia become detached from the otoconial membrane and fall into the semicircular canals before they can dissolve in the endolymph, hence transforming the affected canals into gravity-sensitive organs (9). Cupulolithiasis, in which free-floating endolymph debris adheres to the cupular membrane and renders the canal responsive to gravity (10) has been proposed to account for the remainder of BPPV cases, especially in older patients (10). Clinical signs of cupulolithiasis have been defined as apogeotropic nystagmus, with new maneuvers developed to manage either utricular- or canal-side cupulolithiasis; however, evidence that otoconia adhere strongly or persistently to the cupula is lacking (8).

**Figure 1:**
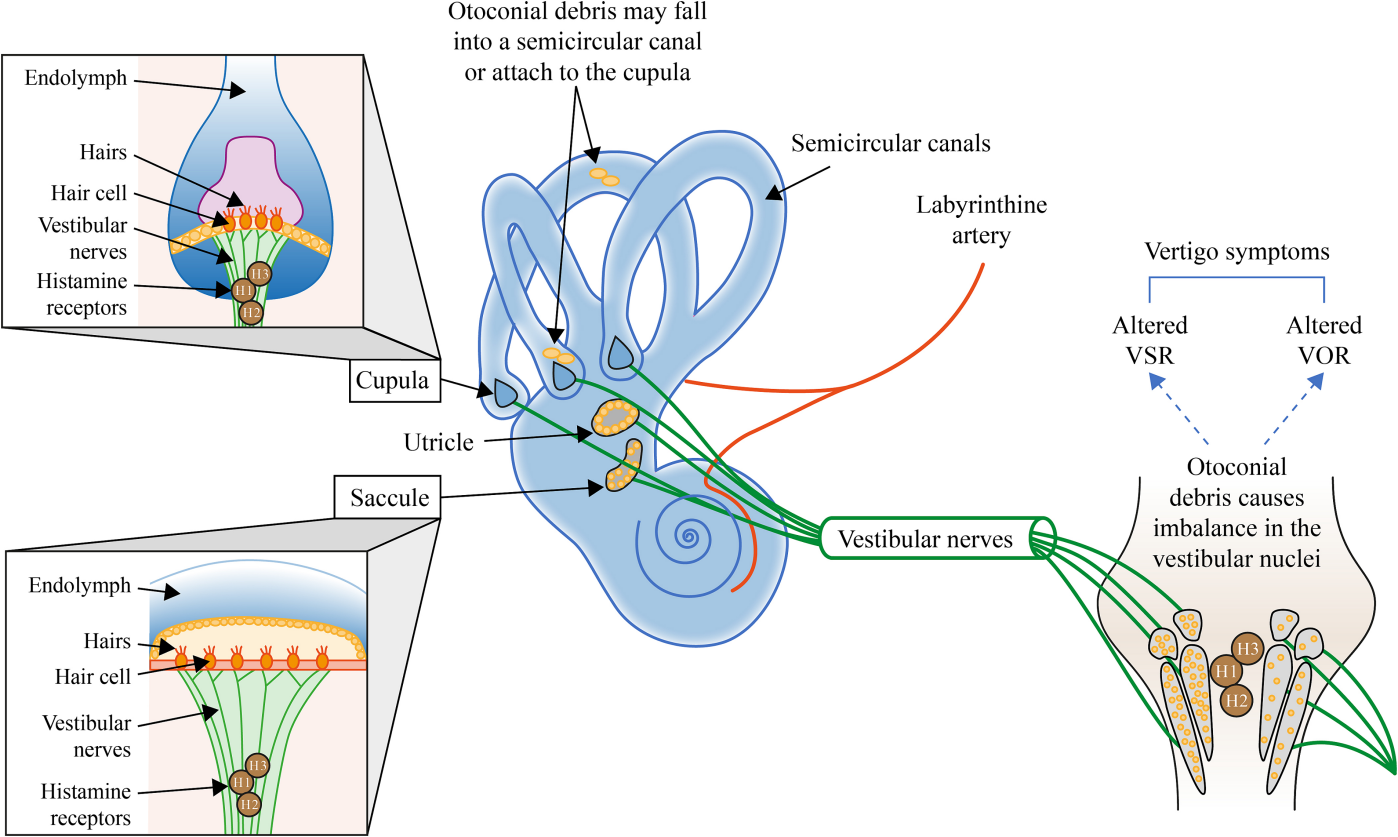
Illustration of the mechanisms involved in BPPV and the potential interventions to address each mechanism. H, Histamine; VOR, Vestibular ocular reflex; and VSR, Vestibulospinal reflex. Otoconial debris may fall into a semicircular canal or attach to the cupula (4). Otoconial debris within the semicircular canals alters the tonic discharge of the affected labyrinth during BPPV, thereby inducing a central adaptation to rebalance the activity of vestibular nuclei to reduce peripheral asymmetry and the symptoms of vertigo (11, 12).

In canalithiasis, the motion of otoconial debris within the semicircular canals due to positional changes of the head alters the tonic discharge of the affected labyrinth during BPPV, thereby inducing asymmetrical resting discharges of the vestibular nuclei on both sides and vertigo symptoms. Reduction of the vertigo symptoms likely requires a central adaptation to rebalance the vestibular nuclei activity (11, 12), see Figure 1.

Many types vestibular pathologies result in symptoms that can occur when the head is kept still (static deficits) or when the head is moving (dynamic deficits). Following acute unilateral peripheral vestibular hypofunction, the static symptoms, usually aggravated by nausea and vomiting, are due to the spontaneous resting activity imbalance between the bilateral vestibular nuclei complexes (13, 14) as well as the strong connection between the vestibular nuclei and neurovegetative centers in the brainstem. Static symptoms are generally fully compensated within a few weeks (15). However, dynamic deficits (the impaired responses when the head is moved) are poorly compensated and are exhibited over a longer time period; in many cases, the VOR does not recover at all (14, 16).

The precise cause of otoconial detachment and moving into the semicircular canals (or attaching to the cupula) is still a subject of study and discussion. The most likely causes include head trauma, aging (through pitting, fissuring, and subsequent fragmentation of otoconia), and increased calcium concentration in the canal endolymph.

It has been hypothesized that otolith detachment in idiopathic BPPV could be secondary to microvascular problems and these can be specifically significant for organs supplied by a terminal-type circulation (17). Ischemia of the neuroepithelium of the utricular macula or semicircular canals may facilitate their degeneration with consequent detachment of otoliths (17). Indeed, vascular risk factors have been reported to be closely related to the occurrence and prognosis of BPPV (18). Considering that the labyrinthine artery of the inner ear is a tiny terminal artery, small vessel lesions that directly damage the tiny arteries of the inner ear may lead to microcirculation disorders, which could cause damage to the blood vessels of the inner ear and lead to shedding of otoliths (18). Patients with hypertension, obesity, and diabetes may also have reduced capillary density that negatively affects microvascular tissue perfusion (19).

There is evidence that BPPV patients have higher levels of oxidative stress than healthy groups. Oxidative stress is a critical factor in microcirculation disturbances through the induction of erythrocyte adhesion to the vascular endothelium and the reduction of erythrocyte deformability (20). It has also been suggested that oxidative stress might play a role in the development of BPPV through its relationship with calcium metabolism and the direct toxic effects of free oxygen radicals, including the triggering of apoptosis (21).

## Risk factors for BPPV

Risk factors for BPPV include head trauma, a prolonged recumbent position, and disorders involving the inner ear; they may also include female gender, age > 65 years, osteoporosis [e.g., 81% of people with BPPV have a decreased bone mass density (10), hypertension, hyperlipidemia, and vitamin D deficiency (5)]. Moreover, type 2 diabetes, mediated by hypertension, hyperglycemia, and hypercholesterolaemia have been noted as risk factors for BPPV (10, 22).

Around one-third of adults >40 years show evidence of vestibular dysfunction (23), with the number, volume and shape of otoconia changing with age and otoconia in the saccule degenerating at a greater rate than those in the utricle (24). Although aging is normally associated with otoconia degeneration, hair cell degeneration, loss of vestibular afferents, and a reduction in the number of cells in the vestibular nuclei, dizziness is not part of normal aging and should be managed (25). Older patients may experience a longer duration of symptoms prior to receiving a diagnosis due to the belief that it is a normal part of aging (26).

## Impact of BPPV

Even though often considered benign in nature, patients with BPPV are markedly limited in their daily activities (5, 14). Almost 86% of patients with BPPV suffer some interruption to their daily activities and lost days at work due to BPPV: 68% reduce their workload; 4% change jobs; and 6% leave their jobs because of the condition (27). Work-related activities tend to be affected more in younger patients (28), while older patients with BPPV experience a greater incidence of falls, depression, and impairments to their daily activities (27, 28).

In the United States, healthcare costs associated with the diagnosis of BPPV alone approach $2 billion per year (27). Much of this can be attributed to the high cost of diagnosis (27), which is estimated to be US$2,000 in the United States, €364 (~US$450) in Spain, RMB 4165.2 Yuan (~US$600) in China, and US$180 in South Korea (5). This healthcare burden is likely to increase as the population ages; in South Korea, the number of hospital visits per 100,000 of general population due to dizziness and vertigo is estimated to increase by around 50% in the next 3 decades (5).

## Diagnosis of BPPV

Diagnosis of BPPV is based on clinical history and the presence and type of nystagmus and rotatory vertigo during positional test. The affected semicircular canal may be identified by the characteristics of the positional nystagmus documented by videonystagmography during the supine roll test, Semont, or Dix-Hallpike test (2).

Up to 20% of cases of positional vertigo may be due to central pathology (29), caused by disease affecting the central nervous system, such as multiple sclerosis, cerebellar disease, and cerebellar stroke, with vestibular migraine being the most common central cause (30). Suspicion of central positional vertigo may be raised with an absence of latency or fatigability of nystagmus, a lack of marked vertigo, pure upbeat or downbeat nystagmus, or as a lack of responsiveness to CRM (29). Therefore, it is recommended that a diagnosis of BPPV can only be made if the supine roll maneuver Semont or Dix-Hallpike tests elicit nystagmus that is consistent with BPPV; any features of the nystagmus not consistent with BPPV should raise suspicion of central pathology and warrant further investigation (29).

There is increasing evidence about the association between vestibular migraine (VM) and BPPV (31). Despite their similarities, BPPV can be differentiated from VM by the direction of the nystagmus and the duration of the symptoms (32). Although there is generally no positional nystagmus in VM, pseudo-BPPV is a complex mix of positional, atypical positional and nonpositional vertigo accompanied by migraine features; the ability to distinguish pseudo-BPPV from other vertigo disease has great clinical significance for treatment (33).

## Management of BPPV

Appropriate canalith repositioning maneuvers (CRMs) to remove the otolith from the semicircular canal, typically by the Epley or Semont maneuvers for posterior canal BPPV (the most frequent type of BPPV) (34), provide relief from symptoms and positional nystagmus in 80% of BPPV patients with a single application (35), and in up to 92% with additional CRMs (36). Other specific maneuvers are designed for the treatment of the less common lateral and anterior canal BPPV (37). CRMs are considered the gold standard first-line treatment (27) and are suitable for most patients, although contraindicated in patients with severe cervical disease, suspected vertebrobasilar disease, unstable cardiovascular disease, or high-grade carotid stenosis (4). As BPPV is not easily resolved in some patients, many different CRMs have been developed to address the numerous variants according to the affected canals (such as Apiani, Gufoni, Zuma, Semont plus, Vanuchi-Asprella, and Yacovino). Even then, not all patients can be cured and it is not unusual that several manoevers are needed to achieve resolution of BPPV.

The outcome of a CRM is dependent on the type of manoever and on the precision of the execution of the manoever. This encompasses a variety of factors including the angle and velocity of the head and timing during each step (38, 39), fluid dynamics, as well as the amount, size, and location of otoconia in the canal. Reports of BPPV recurrence or residual dizziness (RD) after resolution of the initial nystagmus have become more frequent than when CRMs were first introduced (40–42), possibly due to the CRM variant used, the application of the CRM (43), and/or the pathophysiology underlying BPPV itself. For example, it has been demonstrated that CRMs may be less effective in patients with secondary BPPV (27, 44, 45) or lateral canal cupulolithiasis, the latter of which is more common in older patients compared with other types of BPPV (46).

In BPPV, although the positional vertigo may be effectively reduced or eliminated by one or more CRMs, the underlying pathology might be permanent or unresolved, thus increasing the risk for recurrence. There is debate about the potential for and impact of labyrinthine damage in BPPV, but if it exists, it would not be treatable with CRM and could be a cause of ongoing symptoms such as RD, which will be described in more detail below, along with the methods that may be used to evaluate labyrinthine function. So, although CRMs are highly effective in most patients, there is a clear need to develop a comprehensive BPPV treatment plan in which additional BPPV management strategies could be considered for patients in whom a CRM is contraindicated (4); for patients after successful CRM to improve functional recovery, especially in those at risk of RD symptoms; and to manage risk factors associated with BPPV recurrence.

## Additional treatment/management considerations for BPPV

Where labyrinthine function has been permanently changed or reduced, the process of central vestibular compensation recalibrates the part of the brain that controls balance (47). During this process, the intrinsic plasticity of the nervous system is able to reorganize and overcome damage to the peripheral vestibular system through peripheral and central neuronal repair, sensory and behavioral substitution processes, as well as brain remodeling (15). While this vestibular compensation process occurs naturally in most people, the time course varies (15). External factors that may affect the central compensation process include mobilization (rehab) and medication. There is moderate to strong evidence that facilitating vestibular compensation and behavioral adaptation through vestibular rehabilitation therapy (VRT) is a safe, effective management strategy for unilateral peripheral vestibular dysfunction (47, 48). Although vestibular habituation training, a special type of VRT taking several weeks, was applied successfully in BPPV patients, before CRMs were invented, designed, and applied (49), the current management of BPPV patients relies on the different CRMs described in the literature. The popularity of CRMs is partly due to the potential to resolve the vertigo and nystagmus almost immediately. However, VRT may still help in cases of RD after a successful CRM.

Animal studies have shown that histamine receptors (H1, H2, and H3) are upregulated in the central vestibular system during the first few days of vestibular compensation following labyrinthectomy or vestibular nerve section, and the molecular mechanisms by which histamine modulates vestibular function have been clarified and partly elucidated (50). Betahistine, an H3 receptor antagonist and a weak H1 receptor agonist, has been clinically and experimentally shown to facilitate the functional recovery of vestibular functions (50) through reducing spontaneous resting activity imbalance between the bilateral vestibular nuclei complexes (51) and exerting excitatory effects on the vestibular nuclei neurons (52, 53). This is in contrast to vestibular suppressants, such as antihistamines (dimenhydrinate, cinnarizine, meclizine, and promethazine) and benzodiazepines that may provide symptomatic relief but often interfere with the restoration of vestibular functions (47, 54).

The known and proposed mechanisms of action of betahistine and vitamin D supplementation address many of the underlying pathophysiological mechanisms of BPPV and are summarized in Table 1, along with other potential approaches. In some studies, betahistine as an additional therapy to CRM/VRT has been reported to reduce positional vertigo (68–81), although further research is needed to better define patients in whom betahistine offers clinical benefits and to target the underlying cause(s) of their BPPV as well as any associated comorbidities. Although vitamin D insufficiency has been suggested as a possible factor in the pathophysiology of BPPV (5), there is not yet consistent evidence that correction of vitamin D insufficiency cures or prevents recurrence of BPPV.

**Table 1:** Proposed and proven benefits of interventions for BPPV in addition to or after successful CRM^*^.

Benefit	Intervention	Mechanism(s)	Action(s)
Reduce/delay otoconial detachment	Vitamin D supplementation^†^	Maintain low endolymph Ca^2+^ to prevent abnormal otoconia (55).	The biologically active form of vitamin D is involved in the upregulation of epithelial Ca^2+^ channel transporters (55).
	Betahistine^†^	Regulation of intracellular calcium (51).	Action on histamine receptors located in the vestibular periphery (51).
Maintain capacity to dissolve exfoliated otoconia	Vitamin D supplementation^†^	Maintain low endolymph Ca^2+^ to retain capacity to dissolve exfoliated otoconia (55).	The biologically active form of vitamin D is involved in the upregulation of epithelial Ca^2+^ channel transporters (55).
Improve static components	Betahistine^†^	Rebalancing of the vestibular nuclei neurons (51–53).	Improves (reduces) spontaneous resting activity imbalance between the bilateral vestibular nuclei complexes through actions on the histamine H1, H2, and H3 receptors (51). Excitatory effects on the vestibular nuclei neurons (52, 53).
Improve labyrinthine microcirculation	Betahistine^†^	Increases local vestibular blood flow (56).	Inverse agonism at H3 receptor by both betahistine and its major metabolite, aminoethylpyridine (56, 57). Increased release of histamine (direct and indirect via H1 receptors) (58).
	Management of vascular comorbidities^‡^	Vascular comorbidities such as hypertension, dyslipidemia, obesity, and diabetes, may be risk factors for BPPV recurrence (59).	Patients with mild small vessel disease are more likely to suffer a peripheral vestibular disorder (60); their vascular risk factors should be considered in a comprehensive treatment plan, which may include referral to a vascular specialist. High TC levels or hyperlipidemia can cause vascular damage in the inner ear, increasing the risk of BPPV (59). Otolith detachment may be secondary to microvascular dysfunction (17).
Aid central vestibular compensation	Gaze stabilization techniques^‡^	Reduce symptoms of dizziness and vertigo (61).	Improve the gain of the vestibular ocular reflex; improve visual acuity during head movement (61).
	Balance training^‡^	Improved symptom resolution in posterior canalithiasis BPPV (62).	Dissolution and dispersion of otoconial debris and habituation of the pathologic response (62).
	Betahistine^†‡^	Brain arousal, a crucial factor for functional recovery/behavioral adaptation, through general upregulation of histamine (15, 50, 58).^†^	Cerebral H1 receptors that regulate sensorimotor activity (50, 58, 63).
		Increase histamine turnover and release, and modulation of release of other neurotransmitters, particularly GABA that facilitate late-stage vestibular compensation (50, 64, 65).^†^	Antagonistic action on both the presynaptic histamine H3 and postsynaptic histamine H1 receptors (50, 64, 66).
		Promote vestibular compensation of both static and dynamic symptoms: mediation of the asymmetric activation of commissural inhibitory system at circuit level; actively promotes rebalancing of bilateral vestibular systems and vestibular compensation (58).^†^	Partial H1 receptor agonism (47).
		Enhance the rebalancing of the neuronal activity of the vestibular nuclei complexes on both sides (50, 64)^†^ and (67).^‡^	Increased histamine turnover and enhanced histamine release in the CNS (50, 64, 67). H3 receptor antagonism (or H3 receptor inverse agonism) at the level of the secondary vestibular neurons (50, 64, 67).

^*^Current guidelines recommend against the routine treatment of patients with BPPV with vestibular suppressant medications such as antihistamines [dimenhydrinate, cinnarizine, meclizine, promethazine (54), and/or benzodiazepines (27)]. Vestibular suppressant medications (especially benzodiazepines) have the potential for significant harm and may produce drowsiness, cognitive deficits, and interference with driving or operating machinery. Benzodiazepines and antihistamines interfere with central compensation for a vestibular injury (27). ^†^Evidence from animal studies. ^‡^Evidence from clinical studies. BPPV, Benign paroxysmal positional vertigo; Ca, Calcium; CNS, Central nervous system; CRM, Canalith repositioning maneuver; GABA, Gamma-aminobutyric acid; and TC, Total cholesterol.

## Residual dizziness

Resolution of the nystagmus is the key focus of treating BPPV and is achieved in the majority of cases with one or more CRMs (5). However, even after the disappearance of typical vertigo and nystagmus following a successful CRM, some patients report imbalance without positional vertigo, referred to as RD (2). Despite the lack of established diagnostic criteria (55), RD has been defined as a sensation of non-specific dizziness in the absence of positional vertigo and typical nystagmus in patients upon resolution of BPPV (2); it has been reported in 31–61% of patients, with symptoms lasting from a few days to several weeks (2). Of note, RD does not usually meet all the criteria for persistent postural-perceptual dizziness (82). RD induces a very poor quality of life and is incapacitating to most patients who suffer from it (83). Dizziness in the elderly has adverse psychological and social effects including, but not limited to, decreased performance in daily living activities and increased fear of falling (84).

## The pathogenesis of RD

The pathogenesis of RD after resolution of nystagmus with CRM is not fully understood, but a number of hypotheses have been suggested, based on our current understanding of vestibular function.

### Persistence of otoconial debris in the affected semicircular canal

Incomplete repositioning during CRM can result in residual otoconial debris that is sufficient to cause mild positional vertigo or less specific chronic or positional dizziness, but insufficient to deflect the cupula to the degree required to provoke overt nystagmus (58, 85, 86). This suggests a problem in the utriculus in which small particles increase the density of the canal endolymph and interfere with the precision of head motion and tilt detection (including spatial disorientation which might manifest itself as a specific dizziness). In this hypothesis, although liberation maneuvers solve the vertigo, they are unable to resolve the utricular pathology.

### Co-existing vestibular disorders

Many patients with BPPV have a co-existing vestibular disorder. The most common cause of secondary BPPV is head injury, accounting for 7–17% of BPPV cases, followed by viral labyrinthitis or acute unilateral vestibular pathology accounting for up to 15% of BPPV cases (4). Other precipitating conditions that may have occurred previously in the patient’s history, such as Lindsey-Hemingway syndrome, can also cause an imbalance in the vestibular nuclei. In addition, Menière’s disease may predispose patients to intractable BPPV, possibly through hydropically induced damage to the macula of the utricle and saccule or partial obstruction of the membranous labyrinth (87). Compared with patients with uncomplicated BPPV, patients with BPPV and an additional vestibular pathology do not necessarily have a worse prognosis with respect to resolution of positional nystagmus by CRM; however, they are more likely to suffer incomplete resolution of symptoms that require additional vestibular rehabilitation after CRM (88).

### Incomplete vestibular compensation

Residual dizziness after successful CRM in patients with BPPV may also be related to the inability of the central nervous system to re-adjust quickly to a new functional status (11, 68). The sudden resolution of BPPV by CRMs alters the “new equilibrium” achieved, and the inability of central adaptation to promptly restore the pre-existing condition produces the subtle symptoms consistent with RD (11, 12). This delayed central adaptation may be associated with many factors similar to the risk factors for RD, such as the duration of BPPV (68, 89–93) and the patient’s emotional state (68, 91, 92).

### Autonomic dysfunction

Autonomic dysfunction is frequently found in patients with chronic persistent dizziness after excluding other causes (94), such as orthostatic hypotension (95), vascular risk factors (18), and brain atrophy (96). In a study of patients with BPPV who had undergone brain magnetic resonance imaging as research subjects, an association was reported between cerebral small vessel diseases (CSVDs) and RD and recurrence of BPPV (18). It is important that BPPV patients who are prone to RD and/or BPPV recurrence are identified and managed appropriately, specifically older patients with white matter hyperintensity. In addition, it was suggested that BPPV patients with CSVDs should be actively treated at the same time as CRM to reduce the risk of RD (18).

### Somatoform disorder

Anxiety has been demonstrated to play an additional role in dizziness, which may be considered in some cases a somatoform disorder arising from stressful events (91, 97, 98). A recent study in India showed that only 13% of BPPV patients with geotropic nystagmus complained of RD after successful CRM when they were first reassured and counseled about the benign nature of their disorder, suggesting that there is a huge psychological component in post-BPPV syndrome (99). Indeed, patients reporting RD after the successful treatment of BPPV with CRMs tend to be older, more anxious, and/or more stressed (68, 83).

### Low levels of vitamin D

Alterations in calcium metabolism may induce changes in otoconial structure and otolith organ status (93). The biologically active form of vitamin D is involved in the upregulation of epithelial Ca^2+^ channel transporters in the semicircular canal that helps maintain the low endolymph Ca^2+^ concentration in the inner ear. This is vital to prevent the production of abnormal otoconia and maintain the capacity to dissolve exfoliated otoconia (55). Therefore, vitamin D insufficiency could prompt the formation of otoconia or disturb the resorption of dislodged otoconia in the endolymph (55). Animal studies have also suggested a role for vitamin D in vestibular function (6, 100), with vitamin D deficiency associated with otoconial debris and additional dysfunction of otolith organs, thereby contributing to the development of RD (55). Although low serum vitamin D values have been found to correlate with BPPV occurrence/recurrence (55, 101) and abnormal vestibular evoked myogenic potential (VEMP) results (102), only one study has reported an association between low vitamin D levels and RD (93). Vitamin D supplementation might be beneficial in patients with RD and low serum vitamin D levels (103).

### Microcirculation dysfunction

Abnormal cochlear microcirculation has long been considered an important factor in vestibular dysfunction (104). As mentioned above, microvascular problems can contribute to otolith detachment in idiopathic BPPV (17). Studies have also emphasized the role of small vessel disease (SVD) in “unexplained” dizziness in the elderly (60, 105), showing that patients with mild SVD are more likely to suffer a peripheral vestibular disorder (60). We suggest that patients with vascular risk factors should be referred to a specialist.

## Diagnosis and assessment of RD

Although still disputed, utricular function can be measured, to some extent, by subjective visual vertical (SVV), ocular vestibular evoked myogenic potential (o-VEMP), or the unilateral centrifugation test (106). SVV is a test of static otolith function (91, 107); however, the test has a number of challenges and, although a small retrospective study suggested that immediate improvement in SVV scores following CRM may predict BPPV resolution and help identify patients who need greater follow-up (45, 108), its usefulness in assessing RD after BPPV is not clear (11). Ocular-VEMPs (o-VEMPs) are short-latency myogenic potentials produced through the activation of saccular and utricular afferents by sound and vibration, which are an indicator of dynamic otolith function (107). Despite lower accuracy than SVV, a Dizziness Handicap Inventory (DHI) score > 30 or VEMPs impairment have been suggested as markers for the risk of short-term moderate-to-severe RD (108). It is, however, worth mentioning that the clinical relevance of VEMPs for the diagnosis of RD are becoming more controversial.

In the absence of agreed objective measures to assess RD after successful CRM to treat BPPV, most clinical studies have used subjective measures to assess efficacy. Although the DHI and the visual analog scale (VAS) to quantify RD are the most commonly-used measures, neither of these are validated specifically for BPPV or RD. The DHI is a self-assessment inventory of 25 items covering the functional, emotional, and physical impacts on daily life; whereas the VAS has the advantage of being able to distinguish dizziness from vertigo, with reliable results in evaluating RD (91).

## Identifying patients at greater risk for RD

In addition to the aforementioned measurable factors (SVV, o-VEMP, or c-VEMP), several patient or disease characteristics that increase the risk for RD after successful CRM in BPPV patients have been reported. These include:

- Long duration of BPPV before treatment (2, 11, 89, 109).
- Elderly onset age (2, 90, 110–112).
- Female gender (2).
- Physical and psychological comorbidities (2, 82, 90, 92, 111, 113).
- Low vitamin D levels, especially in early-onset female patients (93).

Factors not associated with the occurrence of RD after successful CRM treatment include the affected side, location, or type of semicircular involvement; hyperlipidemia; diabetes; hypertension; and heart disease. Studies have yielded mixed results concerning whether the number of CRM maneuvers required to cure nystagmus in BPPV influences the risk of developing RD (2, 111). In sum, older age and anxiety may be the most important predictors for the occurrence of RD (68); aging has also been reported to contribute to delayed recovery of symptoms associated with RD (46).

## Alternative approaches to the management of RD

Benign paroxysmal positional vertigo, recurrent BPPV, and RD are entities often encountered in clinical practice, especially in the elderly in whom RD may contribute to an increased risk of falls, restrictions in activity, and social and economic burdens (114). It is beyond doubt that CRMs are often successful for eliminating positional vertigo (and are currently the first choice in managing BPPV), but there are many underlying pathologies that not only cause BPPV but also relate to BPPV recurrences and the risk of RD after a successful CRM. A greater understanding of the pathophysiology may help clinicians to develop a BPPV treatment plan and identify the most appropriate therapeutic option for each patient (115, 116). Table 2 summarizes the potential treatment approaches to the management of RD, based on the underlying pathophysiologies described earlier. There is robust evidence for the use of vestibular rehabilitation exercises (1, 61, 62) in optimizing vestibular function, and some evidence for the effect of betahistine on vestibular function (73, 77, 81, 120), and both betahistine (51, 121) and vitamin D supplementation (55, 93) on factors affecting otolith structure and function and inner ear circulation. Table 2 highlights specific patient populations that are at higher risk of RD and should therefore be monitored closely. However, the ability to identify objective markers/measures of RD is essential to accurately and definitively demonstrate clinical benefits of any potential treatment.

**Table 2:** Treatment approaches for managing RD associated with BPPV in specific populations.

Specific patient population	Potential management/treatment option	Underlying mechanism addressed
Patients unable to undergo CRM (4)	Vestibular rehabilitation	Stimulation of natural vestibular compensation processes (49). VR does not reduce the recurrence rate of BPPV but reduces symptoms (1). VR can substitute CRM when spine comorbidities contraindicate CRM (1).
	Betahistine	Improvement of microcirculation and facilitation of otoconial debris clearance (50, 51, 58, 117). Rebalancing of vestibular nuclei and symptomatic relief (50).
**Elderly patients**		
Microcirculation and maintenance of otoconia	Vitamin D supplementation	Older adults are at risk for vitamin D insufficiency due to decreased cutaneous synthesis and dietary intake (118). Epidemiological evidence indicates an association between low vitamin D and diseases that affect the microcirculation (118), such as hypertension and diabetes (59).
	Betahistine	Minimization of otoconial detachment (117). Improvement of microcirculation and facilitation of otoconial debris clearance (50, 51, 58, 117).
Microcirculation	Management of co-morbid cardiometabolic conditions	Vascular comorbidities, such as hypertension, dyslipidemia, obesity and diabetes, may be risk factors for BPPV recurrence (59). Patients with mild small vessel disease are more likely to suffer a peripheral vestibular disorder (60).
Vestibular compensation	Vestibular recovery program	CRM and VR seem to have a synergistic effect in patients with BPPV, especially in elderly patients (1).
	Betahistine	Rebalancing of bilateral vestibular systems and improvement/acceleration of vestibular compensation (51).
Close monitoring	Many elderly patients reporting dizziness may have treatable peripheral vestibular conditions that may be missed if dizziness is solely attributed to aging (60). Close follow-up for signs of BPPV recurrence (45). Patients reporting RD after successful CRM tend to be older and more anxious (68, 99).	
Anxious/depressed patients	Counseling	Patients reporting RD after successful CRM tend to be older and more anxious (68, 99). Otorhinologist/neurotologist/neurologist counseling immediately before and after the CRM has been reported to reduce residual symptoms (99).
Patients with vitamin D deficiency	Vitamin D supplementation in patients with confirmed vitamin D deficiency	Vitamin D plays a critical role in vestibular function (6); decreased serum levels of 25(OH)D were associated with the occurrence and recurrence of BPPV in a Chinese population (100). Minimization of otoconial detachment (117). Older adults are at risk for vitamin D insufficiency due to decreased cutaneous synthesis and dietary intake (118). Epidemiological evidence indicates an association between low vitamin D and diseases that affect the microcirculation (118), such as hypertension and diabetes (59).
Patients with co-existing Meénière’s disease	Betahistine	Rebalancing of the vestibular system; brain arousal favoring sensorimotor activity (47, 50, 58, 63, 64, 67). Expert support for the use of betahistine during the inter-critical phase of MD (66). Benefits of betahistine are improved with higher doses and or when combined with cognitive behavioral therapy (119).

BPPV, Benign paroxysmal positional vertigo; CRM, Canalith repositioning maneuver; MD, Menière’s disease; RD, Residual dizziness; and VR, Vestibular rehabilitation.

## Emerging therapies and future directions

A bibliometric analysis of global research trends in BPPV identified that 40% of publications focused on the treatment of BPPV, 25% on factors influencing the development of BPPV, and 21% on diagnosis; only 7% focused on the hazards associated with BPPV and 5% on its prevention and pathogenesis (122). Interestingly, there is a surge of activity around secondary BPPV caused by inner ear diseases, such as Menière’s disease and sudden deafness (122).

Several nutraceuticals are available with suggested benefits in patients with RD, although the clinical evidence is limited. Using a metabolomics approach, a proprietary polyphenol compound supplementation was reported to affect six metabolites related to RD: 1-methylnicotinamide, anserine, hippurate, lysine, methyl-succinate, and urea (123). However, this was a small study (*n* = 30) and further research is required.

Historically, clinical studies investigating treatments alongside CRM or for the management of RD have not provided conclusive evidence for efficacy due to the lack of a robust measure. Two biomarkers—otolin-1 and elevated red blood cell distribution width (RDW)—have recently been proposed and may prove useful in future clinical studies. Otolin-1 has been identified as a potential biomarker both for the diagnosis of BPPV and in monitoring the effectiveness of betahistine in BPPV patients (124). Otolin-1 is considered an inner ear-specific glycoprotein that can pass the blood-labyrinth barrier and be detected in peripheral blood (124). Elevated RDW is a potential rapid, inexpensive, and readily available laboratory biomarker of RD (20). Elevated RDW promotes platelet activation and aggregation and is a marker of the procoagulant status of red blood cells. It may impair the microcirculation of the labyrinthine artery and increase oxidative stress (20), thus making it a possible indicator of RD. Of note, another assessment—the modified Clinical Test of Sensory Integration and Balance—has been proposed as an objective evaluation of both RD and postural imbalance after a successful CRM for BPPV (125), but it is not specific for RD in BPPV patients.

In terms of our understanding of BPPV pathophysiology, reduced serum levels of superoxide dismutases (SOD) have been associated with a higher risk of BPPV and BPPV recurrence events; possibly due to the role of SOD on inflammatory process. However, further studies are needed to clarify the exact mechanism and whether there is a suitable intervention to ameliorate this process (21). Lastly, a novel mechanism has been proposed for betahistine; animal studies have suggested that the alleviation of BPPV with betahistine may occur through induction of multiple complement 1q/tumor necrosis factor-related proteins and activation of the ERK1/2-AKT/PPARy pathway (126).

**Figure 2:**
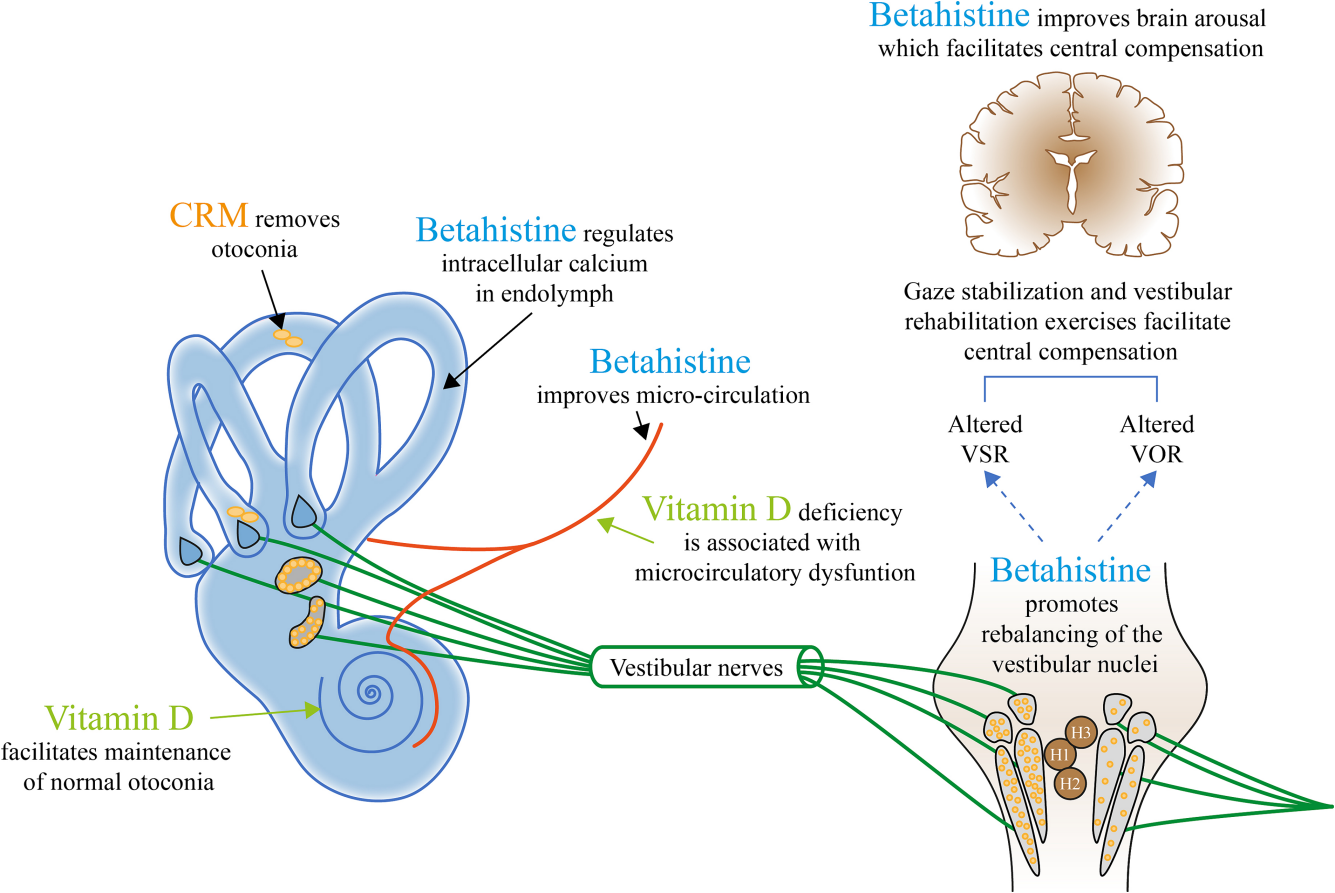
The current potential management approaches that may be considered based on known and proposed mechanisms of BPPV. H, Histamine; VOR, Vestibular ocular reflex; and VSR, Vestibulospinal reflex. Appropriate canalith repositioning maneuvers (CRMs) to remove the otolith from the semicircular canal provide relief from symptoms and positional nystagmus in up to 92% with ≥1 CRMs (36). Vitamin D deficiency is associated with microcirculatory dysfunction (59, 118). The biologically active form of vitamin D is involved in the upregulation of epithelial Ca2+ channel transporters that helps maintain low endolymph Ca^2+^, retain the capacity to dissolve exfoliated otoconia, and prevent abnormal otoconia (55).^†^ Betahistine increases local vestibular blood flow (56)^†^ and is involved in the regulation of intracellular calcium, which helps to reduce/delay otoconial detachment (51).^†^ Betahistine also facilitates central vestibular compensation by enhancing the rebalancing of the neuronal activity of the vestibular nuclei complexes on both sides (50, 64)^†^ and (67).^‡^ Betahistine also improves brain arousal, a crucial factor for functional recovery/behavioral adaptation, through general upregulation of histamine (15, 50, 58).^†^ Gaze stabilization and vestibular rehabilitation exercises facilitate central compensation and reduce symptoms of dizziness and vertigo (1, 61).^‡ †^Evidence from animal studies. ^‡^Evidence from clinical studies.

Research into BPPV and RD should aim to generate a deeper and more accurate theoretical basis on which clinicians can treat the condition through matching treatment modalities to the underlying pathophysiology. Figure 2 provides a general picture highlighting the current potential management approaches that may be considered based on known and proposed mechanisms of BPPV.

## Discussion

**Figure 3:**
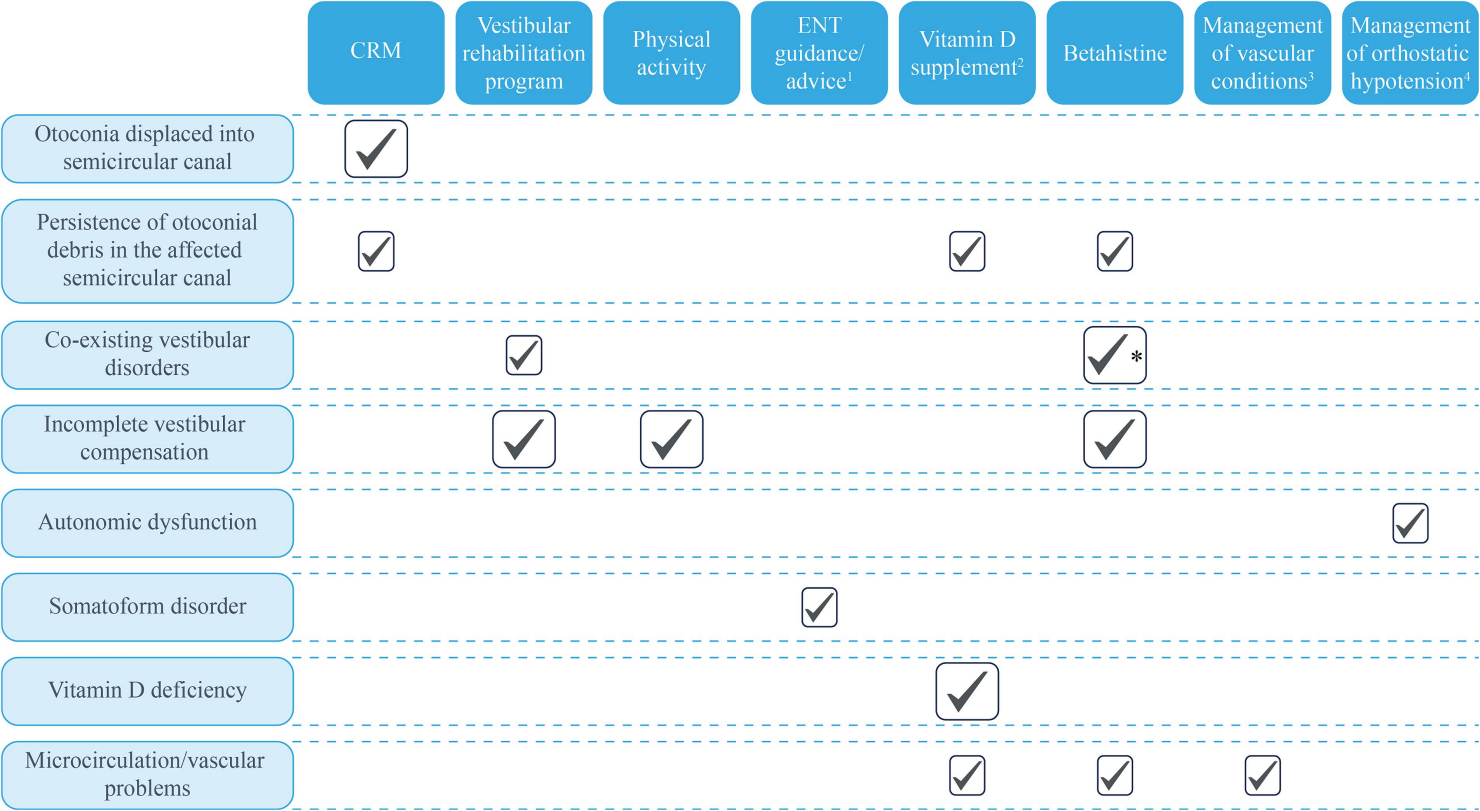
Potential treatment approaches based on known and proposed mechanisms of BPPV. Large ticks represent the mechanisms supported by clinical evidence; small boxes represent potential benefits based on mechanisms with evidence from animal studies or proposed mechanisms. ^1^Explanations and advice from the ENT specialist about what to expect during CRM and the expected trajectory for symptom resolution post-CRM may benefit anxious/stressed patients. ^2^Only for patients with diagnosed vitamin D insufficiency. ^3^Conditions that may affect the microcirculations, such as hypertension and dyslipidemia. ^4^Patients with orthostatic hypotension, vascular risk factors associated with microcirculatory effects, and brain atrophy may be at greater risk of BPPV. ^*^Betahistine is recommended for use in patients with Ménière’s disease. BPPV, Benign paroxysmal positional vertigo; CRM, Canalith repositioning maneuver; and ENT, Ear, nose, and throat.

Benign paroxysmal positional vertigo is a common condition, particularly in older people, associated with decreases in quality of life and interference with daily activities. For the majority of patients, one or more applications of CRM can be successful in relocating the errant otolith debris and restoring normal vestibular function quickly. However, an increasing number of reports indicate that residual symptoms of dizziness are more frequent than previously reported which, although not true vertigo, also reduce quality of life and affect the individual ability to return to normal life. These RD symptoms are more likely to be reported in older patients and those with greater baseline anxiety or stress. As discussed, there are several proposed mechanisms for RD that potential treatments could target. We have suggested a range of additional investigations and treatment options to help clinicians base their management decisions on the underlying pathophysiology for each patient. In particular, these include the use of vestibular habituation therapies and vestibular rehabilitation programs to facilitate vestibular compensation (121); and the potential for betahistine to improve labyrinthine microcirculation and promote vestibular compensation (51, 56); vitamin D supplementation to improve labyrinthine microcirculation in those with a confirmed deficiency (55); and appropriate counseling before and after CRM for patients with a high level of anxiety. On the contrary, vestibular suppressants, such as antihistamines (dimenhydrinate, cinnarizine, meclizine, and promethazine) and/or benzodiazepines, are not recommended for routine use in patients with BPPV (27, 54). For elderly patients, comorbid conditions that may affect labyrinthine microcirculation, including hypertension, dyslipidemia, and diabetes, should be monitored and treated effectively, particularly since BPPV and RD can increase the risk of falls and subsequent morbidity/mortality. A holistic approach to the management of BPPV is key to successful outcomes and reducing the risk of residual dizziness symptoms or BPPV recurrence (Figure 3).

## Author contributions

OÖ: Conceptualization, Methodology, Project administration, Visualization, Writing – original draft, Writing – review & editing. HK: Conceptualization, Methodology, Resources, Visualization, Writing – original draft, Writing – review & editing. LM: Conceptualization, Investigation, Methodology, Resources, Visualization, Writing – original draft, Writing – review & editing. ML: Conceptualization, Investigation, Methodology, Resources, Visualization, Writing – original draft, Writing – review & editing.

## References

[1] Bressi F Vella P Casale M Moffa A Sabatino L Lopez MA. Vestibular rehabilitation in benign paroxysmal positional vertigo: reality or fiction? Int J Immunopathol Pharmacol. (2017) 30:113–22. doi: 10.1177/0394632017709917, PMID: 28485653 PMC5806799

[2] Ke Y Ma X Jing Y Diao T Yu L. Risk factors for residual dizziness in patients with benign paroxysmal positional vertigo after successful repositioning: a systematic review and meta-analysis. Eur Arch Otorrinolaringol. (2022) 279:3237–56. doi: 10.1007/s00405-022-07288-9, PMID: 35218384

[3] Strupp M Bisdorff A Furman J Hornibrook J Jahn K Maire R. Acute unilateral vestibulopathy/vestibular neuritis: diagnostic criteria. J Vestib Res. (2022) 32:389–406. doi: 10.3233/VES-220201, PMID: 35723133 PMC9661346

[4] Palmeri R Kumar A. Benign paroxysmal positional vertigo In: StatPearls. Treasure Island (FL): StatPearls Publishing (2023). Available at: https://www.ncbi.nlm.nih.gov/books/NBK470308/29261987

[5] Kim HJ Park J Kim JS. Update on benign paroxysmal positional vertigo. J Neurol. (2021) 268:1995–2000. doi: 10.1007/s00415-020-10314-7, PMID: 33231724 PMC7684151

[6] Jeong SH Kim JS Kim HJ Choi JY Koo JW Choi KD. Prevention of benign paroxysmal positional vertigo with vitamin D supplementation: a randomized trial. Neurology. (2020) 95:e1117–25. doi: 10.1212/WNL.0000000000010343, PMID: 32759193

[7] Khan S Chang R. Anatomy of the vestibular system: a review. NeuroRehabilitation. (2013) 32:437–43. doi: 10.3233/NRE-130866 23648598

[8] Kalmanson O Foster CA. Cupulolithiasis: a critical reappraisal. OTO Open. (2023) 7:e38. doi: 10.1002/oto2.38, PMID: 36998555 PMC10046726

[9] Rabbitt RD. Semicircular canal biomechanics in health and disease. J Neurophysiol. (2019) 121:732–55. doi: 10.1152/jn.00708.2018, PMID: 30565972 PMC6520623

[10] Yetiser S. Review of the pathology underlying benign paroxysmal positional vertigo. J Int Med Res. (2020) 48:300060519892370. doi: 10.1177/0300060519892370, PMID: 31885315 PMC7605005

[11] Faralli M Lapenna R Giommetti G Pellegrino C Ricci G. Residual dizziness after the first BPPV episode: role of otolithic function and of a delayed diagnosis. Eur Arch Otorrinolaringol. (2016) 273:3157–65. doi: 10.1007/s00405-016-3947-z, PMID: 26926693

[12] Martellucci S Stolfa A Castellucci A Pagliuca G Clemenzi V Terenzi V. Recovery of regular daily physical activities prevents residual dizziness after Canalith repositioning procedures. Int J Environ Res Public Health. (2022) 19:490. doi: 10.3390/ijerph19010490, PMID: 35010750 PMC8744883

[13] Tighilet B Trottier S Mourre C Lacour M. Changes in the histaminergic system during vestibular compensation in the cat. J Physiol. (2006) 573:723–39. doi: 10.1113/jphysiol.2006.107805, PMID: 16613878 PMC1779741

[14] Lacour M Bernard-Demanze L. Interaction between vestibular compensation mechanisms and vestibular rehabilitation therapy: 10 recommendations for optimal functional recovery. Front Neurol. (2014) 5:285. 25610424 10.3389/fneur.2014.00285 PMC4285093

[15] Lacour M Helmchen C Vidal PP. Vestibular compensation: the neuro-otologist's best friend. J Neurol. (2016) 263:54–64. doi: 10.1007/s00415-015-7903-4 PMC4833803 27083885

[16] Darlington CL Smith PF. Molecular mechanisms of recovery from vestibular damage in mammals: recent advances. Prog Neurobiol. (2000) 62:313–25. doi: 10.1016/S0301-0082(00)00002-2, PMID: 10840152

[17] Neri G Filograna Pignatelli GR Pacella A Ortore R Khasawneh L. Recurring paroxysmal positional vertigo: evaluation of the vascular factor. Acta Otorhinolaryngol Ital. (2021) 41:77–83. doi: 10.14639/0392-100X-N0502, PMID: 33746226 PMC7982760

[18] Zang J Jiang X Feng S Zhang H. The influence of cerebral small vessel diseases on the efficacy of repositioning therapy and prognosis of benign paroxysmal positional vertigo. Int J Med Sci. (2022) 19:1227–34. doi: 10.7150/ijms.73080, PMID: 35928725 PMC9346385

[19] Rizzoni D Agabiti-Rosei C Boari GEM Muiesan ML De Ciuceis C. Microcirculation in hypertension: a therapeutic target to prevent cardiovascular disease? J Clin Med. (2023) 12:4892. doi: 10.3389/fneur.2014.00285, PMID: 37568294 PMC10419740

[20] Xie KH Chen LC Liu LL Su CY Li H Liu RN. Elevated red cell distribution width predicts residual dizziness in patients with benign paroxysmal positional vertigo. Front Neurol. (2022) 13:857133. doi: 10.3389/fneur.2022.857133, PMID: 36119686 PMC9477442

[21] Şahin E Deveci İ Dinç ME Özker BY Biçer C Erel Ö. Oxidative status in patients with benign paroxysmal positional Vertigo. J Int Adv Otol. (2018) 14:299–303. doi: 10.5152/iao.2018.4756, PMID: 30256204 PMC6354457

[22] Stapleton PA Goodwill AG James ME Brock RW Frisbee JC. Hypercholesterolemia and microvascular dysfunction: interventional strategies. J Inflamm. (2010) 7:54. doi: 10.1186/1476-9255-7-54, PMID: 21087503 PMC2996379

[23] Wagner AR Akinsola O Chaudhari AMW Bigelow KE Merfeld DM. Measuring vestibular contributions to age-related balance impairment: a review. Front Neurol. (2021) 12:635305. doi: 10.3389/fneur.2021.635305, PMID: 33633678 PMC7900546

[24] Ji L Zhai S. Aging and the peripheral vestibular system. J Otolaryngol. (2018) 13:138–40. doi: 10.1016/j.joto.2018.11.006, PMID: 30671091 PMC6335476

[25] Zalewski CK. Aging of the human vestibular system. Semin Hear. (2015) 36:175–96. doi: 10.1055/s-0035-1555120, PMID: 27516717 PMC4906308

[26] Furman JM Raz Y Whitney SL. Geriatric vestibulopathy assessment and management. Curr Opin Otolaryngol Head Neck Surg. (2010) 18:386–91. doi: 10.1097/MOO.0b013e32833ce5a6, PMID: 20613528 PMC4879828

[27] Bhattacharyya N Gubbels SP Schwartz SR Edlow JA El-Kashlan H Fife T. Clinical practice guideline: benign paroxysmal positional vertigo (Update). Otolaryngol Head Neck Surg. (2017) 156:S1-S47. doi: 10.1177/0194599816689667 28248609

[28] Kao CL Hsieh WL Chern CM Chen LK Lin MH Chan RC. Clinical features of benign paroxysmal positional vertigo (BPPV) in Taiwan: differences between young and senior age groups. Arch Gerontol Geriatr. (2009) 49:S50–4. doi: 10.1016/S0167-4943(09)70014-7, PMID: 20005428

[29] Power L Murray K Bullus K Drummond KJ Trost N Szmulewicz DJ. Central conditions mimicking benign paroxysmal positional Vertigo: a case series. J Neurol Phys Ther. (2019) 43:186–91. doi: 10.1097/NPT.0000000000000276 31136448

[30] Koohi N Male AJ Kaski D. Acute positional vertigo in the emergency department-peripheral vs. central positional nystagmus. Front Neurol. (2023) 14:1266778. doi: 10.3389/fneur.2023.1266778, PMID: 37869150 PMC10585259

[31] Power L Murray K Szmulewicz DJ. Characteristics of assessment and treatment in benign paroxysmal positional vertigo (BPPV). J Vestib Res. (2020) 30:55–62. doi: 10.3233/VES-190687 31839619 PMC9249279

[32] von Brevern M Radtke A Clarke AH Lempert T. Migrainous vertigo presenting as episodic positional vertigo. Neurology. (2004) 62:469–72. doi: 10.1212/01.WNL.0000106949.55346.CD 14872034

[33] Yu J Yu Q Guan B Lu Y Chen C Yu S. Pseudo-benign paroxysmal positional Vertigo: a retrospective study and case report. Front Neurol. (2020) 11:187. doi: 10.3389/fneur.2020.00187 32265827 PMC7105806

[34] Califano L Salafia F Mazzone S Melillo MG Califano M. Anterior canal BPPV and apogeotropic posterior canal BPPV: two rare forms of vertical canalolithiasis. Acta Otorhinolaryngol Ital. (2014) 34:189–97. PMID: 24882928 PMC4035840

[35] von Brevern M Seelig T Radtke A Tiel-Wilck K Neuhauser H Lempert T. Short-term efficacy of Epley's manoeuvre: a double-blind randomised trial. J Neurol Neurosurg Psychiatry. (2006) 77:980–2. doi: 10.1136/jnnp.2005.085894, PMID: 16549410 PMC2077628

[36] Gordon CR Gadoth N. Repeated vs single physical maneuver in benign paroxysmal positional vertigo. Acta Neurol Scand. (2004) 110:166–9. doi: 10.1111/j.1600-0404.2004.00296.x, PMID: 15285773

[37] Bhandari A Bhandari R Kingma H Strupp M. Diagnostic and therapeutic maneuvers for anterior canal BPPV canalithiasis: three-dimensional simulations. Front Neurol. (2021) 12:740599. doi: 10.3389/fneur.2021.740599, PMID: 34630309 PMC8497794

[38] Bhandari A Bhandari R Kingma H Zuma EMF Strupp M. Three-dimensional simulations of six treatment maneuvers for horizontal canal benign paroxysmal positional vertigo canalithiasis. Eur J Neurol. (2021) 28:4178–83. doi: 10.1111/ene.15044, PMID: 34339551

[39] Bhandari R Bhandari A Kingma H Berg RV. Large variability of head angulation during the Epley maneuver: use of a head-mounted guidance system with visual feedback to improve outcomes. J Int Adv Otol. (2023) 19:234–41. doi: 10.5152/iao.2023.22969, PMID: 37272642 PMC10331711

[40] Celis-Aguilar E Mayoral-Flores HO Torrontegui-Zazueta LA Medina-Cabrera CA León-Leyva IC Dehesa-López E. Effectiveness of Brandt Daroff, Semont and Epley maneuvers in the treatment of benign paroxysmal positional Vertigo: a randomized controlled clinical trial. Indian J Otolaryngol Head Neck Surg. (2022) 74:314–21. doi: 10.1007/s12070-021-02516-w, PMID: 36213465 PMC9535051

[41] Cetin YS Ozmen OA Demir UL Kasapoglu F Basut O Coskun H. Comparison of the effectiveness of Brandt-Daroff vestibular training and Epley Canalith repositioning maneuver in benign paroxysmal positional vertigo long term result: a randomized prospective clinical trial. Pak J Med Sci. (2018) 34:558–63. doi: 10.12669/pjms.343.14786, PMID: 30034415 PMC6041543

[42] Çetin YS Çağaç A Düzenli U Bozan N Elasan S. Residual dizziness in elderly patients after benign paroxysmal positional vertigo. ORL J Otorhinolaryngol Relat Spec. (2022) 84:122–9. doi: 10.1159/000516961, PMID: 34237746

[43] Hougaard DD Valsted SH Bruun NH Bech MW Talebnasab MH. Seven years of experience with treatment of benign paroxysmal positional vertigo with a mechanical rotational chair. Front Neurol. (2022) 13:981216. doi: 10.3389/fneur.2022.981216, PMID: 36090886 PMC9453247

[44] Kansu L Avci S Yilmaz I Ozluoglu LN. Long-term follow-up of patients with posterior canal benign paroxysmal positional vertigo. Acta Otolaryngol. (2010) 130:1009–12. doi: 10.3109/00016481003629333, PMID: 20297928

[45] Little CCSZ Campo M Gurley J Hujsak B Cosetti MK Kelly J. Immediate improvement in subjective visual vertical and disequilibrium predicts resolution of benign paroxysmal positional Vertigo following single Canalith repositioning maneuver. Otol Neurotol Open. (2022) 2:e014. doi: 10.1097/ONO.0000000000000014, PMID: 38516626 PMC10950152

[46] Yetiser S Salturk Z. A review of the quality of life after therapeutic maneuvers in patients with benign paroxysmal positional Vertigo. Iran J Otorhinolaryngol. (2021) 33:339–46. doi: 10.22038/IJORL.2021.55574.2912, PMID: 35223650 PMC8829782

[47] Chen ZP Zhang XY Peng SY Yang ZQ Wang YB Zhang YX. Histamine H1 receptor contributes to vestibular compensation. J Neurosci. (2019) 39:420–33. doi: 10.1523/JNEUROSCI.1350-18.2018, PMID: 30413645 PMC6335742

[48] Hillier SL McDonnell M. Vestibular rehabilitation for unilateral peripheral vestibular dysfunction. Cochrane Database Syst Rev. (2011) 2:Cd005397. doi: 10.1002/14651858.CD005397.pub3 21328277

[49] Norré ME. Rationale of rehabilitation treatment for vertigo. Am J Otolaryngol. (1987) 8:31–5. doi: 10.1016/S0196-0709(87)80016-9, PMID: 3495192

[50] Lacour M. Betahistine treatment in managing vertigo and improving vestibular compensation: clarification. J Vestib Res. (2013) 23:139–51. doi: 10.3233/VES-130496, PMID: 24177346

[51] Lacour M Sterkers O. Histamine and betahistine in the treatment of vertigo: elucidation of mechanisms of action. CNS Drugs. (2001) 15:853–70. doi: 10.2165/00023210-200115110-00004 11700150

[52] Yu L Zhang XY Cao SL Peng SY Ji DY Zhu JN. Na(+) -ca(2+) exchanger, leak K(+) channel and hyperpolarization-activated cyclic nucleotide-Gated Channel Comediate the histamine-induced excitation on rat inferior vestibular nucleus neurons. CNS Neurosci Ther. (2016) 22:184–93. doi: 10.1111/cns.12451, PMID: 26387685 PMC6492809

[53] Zhang XY Yu L Zhuang QX Peng SY Zhu JN Wang JJ. Postsynaptic mechanisms underlying the excitatory action of histamine on medial vestibular nucleus neurons in rats. Br J Pharmacol. (2013) 170:156–69. doi: 10.1111/bph.12256, PMID: 23713466 PMC3764857

[54] Di Mizio G Marcianò G Palleria C Muraca L Rania V Roberti R. Drug–drug interactions in vestibular diseases, clinical problems, and medico-legal implications. Int J Environ Res Public Health. (2021) 18:12936. doi: 10.3390/ijerph182412936, PMID: 34948545 PMC8701970

[55] Wu J Jiang CY Bai YX Xu Q Sun XH Pan H. Effect of the serum 25-hydroxyvitamin D level on risk for short-term residual dizziness after successful repositioning in benign paroxysmal positional vertigo stratified by sex and onset age. Front Neurol. (2023) 14:1144958. doi: 10.3389/fneur.2023.1144958, PMID: 37064183 PMC10102369

[56] Dziadziola JK Laurikainen EL Rachel JD Quirk WS. Betahistine increases vestibular blood flow. Otolaryngol Head Neck Surg. (1999) 120:400–5. doi: 10.1016/S0194-5998(99)70283-4, PMID: 10064646

[57] Ihler F Bertlich M Sharaf K Strieth S Strupp M Canis M. Betahistine exerts a dose-dependent effect on cochlear stria vascularis blood flow in guinea pigs in vivo. PLoS One. (2012) 7:e39086. doi: 10.1371/journal.pone.0039086, PMID: 22745706 PMC3380058

[58] Benny R. Expert opinions regarding neuro-microcirculatory, vestibular and labyrinthine dynamics in benign paroxysmal positional vertigo. Int J Res Med Sci. (2022) 10:796–800. doi: 10.18203/2320-6012.ijrms20220541

[59] Chen J Zhang S Cui K Liu C. Risk factors for benign paroxysmal positional vertigo recurrence: a systematic review and meta-analysis. J Neurol. (2021) 268:4117–27. doi: 10.1007/s00415-020-10175-0, PMID: 32839838

[60] Cerchiai N Mancuso M Navari E Giannini N Casani AP. Aging with cerebral small vessel disease and dizziness: the importance of undiagnosed peripheral vestibular disorders. Front Neurol. (2017) 8:241. doi: 10.3389/fneur.2017.00241, PMID: 28626444 PMC5454069

[61] Meldrum D Jahn K. Gaze stabilisation exercises in vestibular rehabilitation: review of the evidence and recent clinical advances. J Neurol. (2019) 266:11–8. doi: 10.1007/s00415-019-09459-x, PMID: 31385017

[62] Teixido M Casserly R Melley LE. Lateral modified Brandt-Daroff exercises: a novel home treatment technique for horizontal canal BPPV. J Int Adv Otol. (2021) 17:52–7. doi: 10.5152/iao.2020.9452, PMID: 33605222 PMC7901422

[63] Lacour M van de Heyning PH Novotny M Tighilet B. Betahistine in the treatment of Ménière's disease. Neuropsychiatr Dis Treat. (2007) 3:429–40. PMID: 19300572 PMC2655085

[64] Lacour M Tighilet B. Vestibular compensation in the cat: the role of the histaminergic system. Acta Otolaryngol Suppl. (2000) 544:15–8. doi: 10.1080/000164800750044434, PMID: 10904796

[65] Casani AP Navari E Guidetti G Lacour M. Good clinical approach: Delphi consensus for the use of Betahistine in Menière's disease. Int J Otolaryngol. (2018) 2018:1–11. doi: 10.1155/2018/5359208 PMC6222235 30498513

[66] Casani AP Guidetti G Schoenhuber R. Report from a consensus conference on the treatment of Ménière's disease with betahistine: rationale, methodology and results. Acta Otorhinolaryngol Ital. (2018) 38:460–7. doi: 10.14639/0392-100X-2035, PMID: 30498275 PMC6265668

[67] Redon C Lopez C Bernard-Demanze L Dumitrescu M Magnan J Lacour M. Betahistine treatment improves the recovery of static symptoms in patients with unilateral vestibular loss. J Clin Pharmacol. (2011) 51:538–48. doi: 10.1177/0091270010369241, PMID: 20940335

[68] Martellucci S Pagliuca G de Vincentiis M Greco A De Virgilio A Nobili Benedetti FM. Features of residual dizziness after canalith repositioning procedures for benign paroxysmal positional Vertigo. Otolaryngol Head Neck Surg. (2016) 154:693–701. doi: 10.1177/0194599815627624, PMID: 26861236

[69] Pérez-Garrigues H Kuessner D Benecke H. Patient baseline characteristics in an open-label multinational study of betahistine in recurrent peripheral vestibular vertigo: the OSVaLD study. Curr Med Res Opin. (2007) 23:2753–61. doi: 10.1185/03007X233016, PMID: 17910803

[70] Benecke H Pérez-Garrigues H Bin Sidek D Uloziene I Sondag E Theeuwes A. Effects of betahistine on patient-reported outcomes in routine practice in patients with vestibular vertigo and appraisal of tolerability: experience in the OSVaLD study. Int Tinnitus J. (2010) 16:14–24. PMID: 21609908

[71] Parfenov VA Golyk VA Matsnev EI Morozova SV Melnikov OA Antonenko LM. Effectiveness of betahistine (48 mg/day) in patients with vestibular vertigo during routine practice: the VIRTUOSO study. PLoS One. (2017) 12:e0174114. doi: 10.1371/journal.pone.0174114, PMID: 28358888 PMC5373561

[72] Sanchez-Vanegas G Castro-Moreno C Buitrago D. Betahistine in the treatment of peripheral vestibular Vertigo: results of a real-life study in primary care. Ear Nose Throat J. (2020) 99:356–60. doi: 10.1177/0145561319849946, PMID: 31111729

[73] Sayin I Koç RH Temirbekov D Gunes S Cirak M Yazici ZM. Betahistine add-on therapy for treatment of subjects with posterior benign paroxysmal positional vertigo: a randomized controlled trial. Braz J Otorhinolaryngol. (2022) 88:421–6. doi: 10.1016/j.bjorl.2020.07.011, PMID: 32978116 PMC9422698

[74] Kaur J Shamanna K. Management of Benign Paroxysmal Positional Vertigo: a comparative study between Epleys Manouvre and Betahistine. Int Tinnitus J. (2017) 21:30–4. doi: 10.5935/0946-5448.20170007, PMID: 28723599

[75] Guneri EA Kustutan O. The effects of betahistine in addition to epley maneuver in posterior canal benign paroxysmal positional vertigo. Otolaryngol Head Neck Surg. (2012) 146:104–8. doi: 10.1177/0194599811419093, PMID: 21852389

[76] Maslovara S Soldo SB Puksec M Balaban B Penavic IP. Benign paroxysmal positional vertigo (BPPV): influence of pharmacotherapy and rehabilitation therapy on patients' recovery rate and life quality. NeuroRehabilitation. (2012) 31:435–41. doi: 10.3233/NRE-2012-00814, PMID: 23232168

[77] Stambolieva K Angov G. Effect of treatment with betahistine dihydrochloride on the postural stability in patients with different duration of benign paroxysmal positional vertigo. Int Tinnitus J. (2010) 16:32–6. PMID: 21609911

[78] Cavaliere M Mottola G Iemma M. Benign paroxysmal positional vertigo: a study of two manoeuvres with and without betahistine. Acta Otorhinolaryngol Ital. (2005) 25:107–12. PMID: 16116833 PMC2639876

[79] Ugurlu BEM Ozkurt FE Sapci T Gursel AO. Comparison of the effects of Betahistine Dihydrochloride and Brandt-Daroff exercises in addition to Epley maneuver in the treatment of benign paroxysmal positional Vertigo. Int Adv Otol. (2012) 8:45–50.

[80] Muhammad TAE Habib M Tayyab M Arshad M Samin KA. Comparison of effectiveness of Epley’s maneuver only and Epley’s maneuver plus Betahistinein the Management of Benign Paroxysmal Positional Vertigo. PJMHS. (2021) 15:1254–6.

[81] Li W Sun J Zhao Z Xu J Wang H Ding R. Efficacy of Epley's maneuver plus betahistine in the management of PC-BPPV: a systematic review and meta-analysis. Medicine (Baltimore). (2023) 102:e33421. doi: 10.1097/MD.0000000000033421, PMID: 37000080 PMC10063308

[82] Staab JP Eckhardt-Henn A Horii A Jacob R Strupp M Brandt T. Diagnostic criteria for persistent postural-perceptual dizziness (PPPD): consensus document of the committee for the classification of vestibular disorders of the Bárány society. J Vestib Res. (2017) 27:191–208. doi: 10.3233/VES-170622, PMID: 29036855 PMC9249299

[83] Biswas ADN. Role of betahistine in the management of vertigo. Ann Otol Neurotol. (2018) 1:51–7. doi: 10.1055/s-0038-1676875

[84] Vanspauwen R. Dizziness and (fear of) falling in the elderly: a few facts. J Int Adv Otol. (2018) 14:1–2. doi: 10.5152/iao.2018.0201815, PMID: 29764772 PMC6354480

[85] von Brevern M Schmidt T Schönfeld U Lempert T Clarke AH. Utricular dysfunction in patients with benign paroxysmal positional vertigo. Otol Neurotol. (2006) 27:92–6. doi: 10.1097/01.mao.0000187238.56583.9b 16371853

[86] Inukai K Koizuka I Takahashi S. Investigation into dizziness before and after Epley's maneuver for benign paroxysmal positional vertigo using stabilometry. Auris Nasus Larynx. (2007) 34:15–7. doi: 10.1016/j.anl.2006.09.018, PMID: 17118595

[87] Gross EM Ress BD Viirre ES Nelson JR Harris JP. Intractable benign paroxysmal positional vertigo in patients with Meniere's disease. Laryngoscope. (2000) 110:655–9. doi: 10.1097/00005537-200004000-00022, PMID: 10764014

[88] Pollak L Davies RA Luxon LL. Effectiveness of the particle repositioning maneuver in benign paroxysmal positional vertigo with and without additional vestibular pathology. Otol Neurotol. (2002) 23:79–83. doi: 10.1097/00129492-200201000-00018, PMID: 11773852

[89] Seok JI Lee HM Yoo JH Lee DK. Residual dizziness after successful repositioning treatment in patients with benign paroxysmal positional vertigo. J Clin Neurol. (2008) 4:107–10. doi: 10.3988/jcn.2008.4.3.107, PMID: 19513312 PMC2686873

[90] Teggi R Giordano L Bondi S Fabiano B Bussi M. Residual dizziness after successful repositioning maneuvers for idiopathic benign paroxysmal positional vertigo in the elderly. Eur Arch Otorrinolaringol. (2011) 268:507–11. doi: 10.1007/s00405-010-1422-9, PMID: 21069369

[91] Giommetti G Lapenna R Panichi R Mobaraki PD Longari F Ricci G. Residual dizziness after successful repositioning maneuver for idiopathic benign paroxysmal positional Vertigo: a review. Audiol Res. (2017) 7:178. doi: 10.4081/audiores.2017.178, PMID: 28603599 PMC5452628

[92] Faralli M Ricci G Ibba MC Crognoletti M Longari F Frenguelli A. Dizziness in patients with recent episodes of benign paroxysmal positional vertigo: real otolithic dysfunction or mental stress? J Otolaryngol Head Neck Surg. (2009) 38:375–80. PMID: 19476771

[93] Wu Y Han K Han W Fan Z Zhou M Lu X. Low 25-Hydroxyvitamin D levels are associated with residual dizziness after successful treatment of benign paroxysmal positional Vertigo. Front Neurol. (2022) 13:915239. doi: 10.3389/fneur.2022.915239, PMID: 35812091 PMC9256914

[94] Lee H Kim HA. Autonomic dysfunction in chronic persistent dizziness. J Neurol Sci. (2014) 344:165–70. doi: 10.1016/j.jns.2014.06.048, PMID: 25012479

[95] Kim HA Lee H. Autonomic dysfunction as a possible cause of residual dizziness after successful treatment in benign paroxysmal positional vertigo. Clin Neurophysiol. (2014) 125:608–14. doi: 10.1016/j.clinph.2013.08.008, PMID: 24045026

[96] Cha WW Song K Yu IK Choi MS Chang DS Cho CS. Magnetic resonance imaging predicts chronic dizziness after benign paroxysmal positional vertigo. Am J Otolaryngol. (2017) 38:428–32. doi: 10.1016/j.amjoto.2017.04.001, PMID: 28390809

[97] Brandt T. Phobic postural vertigo. Neurology. (1996) 46:1515–9. doi: 10.1212/WNL.46.6.1515 8649539

[98] Huppert D Strupp M Rettinger N Hecht J Brandt T. Phobic postural vertigo--a long-term follow-up (5 to 15 years) of 106 patients. J Neurol. (2005) 252:564–9. doi: 10.1007/s00415-005-0699-x 15742115

[99] Biswas A Dutta N. Post-BPPV syndrome: our experience in a specialized neurotology clinic in Kolkata. Ann Otol Neurotol. (2019) 2:01–9. doi: 10.1055/s-0039-1695666

[100] Ding J Liu L Kong WK Chen XB Liu X. Serum levels of 25-hydroxy vitamin D correlate with idiopathic benign paroxysmal positional vertigo. Biosci Rep. (2019) 39:BSR20190142. doi: 10.1042/BSR20190142, PMID: 30962270 PMC6488856

[101] AlGarni MA Mirza AA Althobaiti AA Al-Nemari HH Bakhsh LS. Association of benign paroxysmal positional vertigo with vitamin D deficiency: a systematic review and meta-analysis. Eur Arch Otorrinolaringol. (2018) 275:2705–11. doi: 10.1007/s00405-018-5146-6, PMID: 30302575

[102] Sanyelbhaa H Sanyelbhaa A. Vestibular-evoked myogenic potentials and subjective visual vertical testing in patients with vitamin D deficiency/insufficiency. Eur Arch Otorrinolaringol. (2015) 272:3233–9. doi: 10.1007/s00405-014-3395-6, PMID: 25411075

[103] Fan Z Hu Z Han W Lu X Liu X Zhou M. High serum levels of Otolin-1 in patients with benign paroxysmal positional Vertigo predict recurrence. Front Neurol. (2022) 13:841677. doi: 10.3389/fneur.2022.841677, PMID: 35359660 PMC8963966

[104] Shi X. Physiopathology of the cochlear microcirculation. Hear Res. (2011) 282:10–24. doi: 10.1016/j.heares.2011.08.006, PMID: 21875658 PMC3608480

[105] Kaski D Rust HM Ibitoye R Arshad Q Allum JHJ Bronstein AM. Theoretical framework for "unexplained" dizziness in the elderly: the role of small vessel disease. Prog Brain Res. (2019) 248:225–40. doi: 10.1016/bs.pbr.2019.04.009, PMID: 31239134

[106] Saxena S Patel B Roy R Swami H Singh SK Goyal S. Role of subjective visual vertical in patients with posterior canal benign paroxysmal positional vertigo as a prognostic marker after canalith repositioning maneuver. J Otolaryngol. (2022) 17:111–5. doi: 10.1016/j.joto.2022.03.002, PMID: 35847572 PMC9270559

[107] Taylor RL Welgampola MS. Otolith function testing. Adv Otorhinolaryngol. (2019) 82:47–55. doi: 10.1159/000490271 30947185

[108] Jiang CY Wu J Shu L Sun XH Pan H Xu Q. Clinical and cVEMP evaluation predict short-term residual dizziness after successful repositioning in benign paroxysmal positional Vertigo. Front Med. (2022) 9:881307. doi: 10.3389/fmed.2022.881307, PMID: 35685419 PMC9170995

[109] Teggi R Quaglieri S Gatti O Benazzo M Bussi M. Residual dizziness after successful repositioning maneuvers for idiopathic benign paroxysmal positional vertigo. ORL J Otorhinolaryngol Relat Spec. (2013) 75:74–81. doi: 10.1159/000350255, PMID: 23774304

[110] Sloane PD Baloh RW. Persistent dizziness in geriatric patients. J Am Geriatr Soc. (1989) 37:1031–8. doi: 10.1111/j.1532-5415.1989.tb06916.x, PMID: 2809049

[111] Vaduva C Estéban-Sánchez J Sanz-Fernández R Martín-Sanz E. Prevalence and management of post-BPPV residual symptoms. Eur Arch Otorrinolaringol. (2018) 275:1429–37. doi: 10.1007/s00405-018-4980-x, PMID: 29687182

[112] Fu W He F Bai Y Wang Y Wei D Shi Y. Assessment of residual dizziness after successful canalith repositioning maneuvre in benign paroxysmal positional vertigo patients: a questionnaire-based study. Eur Arch Otorrinolaringol. (2023) 280:137–41. doi: 10.1007/s00405-022-07474-9, PMID: 35727415

[113] Fu W He F Bai Y An X Shi Y Han J. Risk factors of residual dizziness after successful treatment for benign paroxysmal positional Vertigo in middle-aged and older adults. Front Neurol. (2022) 13:850088. doi: 10.3389/fneur.2022.850088, PMID: 36176560 PMC9514231

[114] Silva CN Ribeiro KM Freitas RV Ferreira LM Guerra RO. Vertiginous symptoms and objective measures of postural balance in elderly people with benign paroxysmal positional vertigo submitted to the Epley maneuver. Int Arch Otorhinolaryngol. (2016) 20:61–8. doi: 10.1055/s-0035-1565915, PMID: 26722348 PMC4687998

[115] van de Berg R Kingma H. History taking in non-acute vestibular symptoms: a 4-step approach. J Clin Med. (2021) 10:5726. doi: 10.3390/jcm10245726 34945023 PMC8703413

[116] Lee S-H Kim JS. Benign Paroxysmal Positional Vertigo. J Clin Neurol. (2010) 6:51–63. doi: 10.3988/jcn.2010.6.2.51 20607044 PMC2895225

[117] Libonati GA Leone A Martellucci S Gallo A Albera R Lucisano S. Prevention of recurrent benign paroxysmal positional Vertigo: the role of combined supplementation with vitamin D and antioxidants. Audiol Res. (2022) 12:445–56. doi: 10.3390/audiolres12040045, PMID: 36004953 PMC9404917

[118] Meehan M Penckofer S. The role of vitamin D in the aging adult. J Aging Gerontol. (2014) 2:60–71. doi: 10.12974/2309-6128.2014.02.02.1, PMID: 25893188 PMC4399494

[119] Adrion C Fischer CS Wagner J Gürkov R Mansmann U Strupp M. Efficacy and safety of betahistine treatment in patients with Meniere's disease: primary results of a long term, multicentre, double blind, randomised, placebo controlled, dose defining trial (BEMED trial). BMJ. (2016) 352:h6816. doi: 10.1136/bmj.h6816 26797774 PMC4721211

[120] Jalali MM Gerami H Saberi A Razaghi S. The impact of Betahistine versus Dimenhydrinate in the resolution of residual dizziness in patients with benign paroxysmal positional Vertigo: a randomized clinical trial. Ann Otol Rhinol Laryngol. (2020) 129:434–40. doi: 10.1177/0003489419892285, PMID: 31810393

[121] Teixeira LJ Machado JN. Maneuvers for the treatment of benign positional paroxysmal vertigo: a systematic review. Braz J Otorhinolaryngol. (2006) 72:130–8. doi: 10.1016/S1808-8694(15)30046-X, PMID: 16917565 PMC9445677

[122] Hu Y Lu Y Wang S Quan X Ren Y Rong K. Global research trends in benign paroxysmal positional vertigo: a bibliometric analysis. Front Neurol. (2023) 14:1204038. doi: 10.3389/fneur.2023.1204038, PMID: 37333008 PMC10272773

[123] Casani AP Albera R Piras C Albera A Noto A Ducci N. Clinical efficacy and metabolomics modifications induced by polyphenol compound supplementation in the treatment of residual dizziness following Semont maneuver in benign paroxysmal positional Vertigo (BPPV) of the posterior Semicircular Canal (PSC): preliminary results. Meta. (2024) 14:86. doi: 10.3390/metabo14020086 PMC10890690 38392978

[124] Karaer IC UA Akalin Y (2023). Otolin-1 as a biomarker for the evaluation of the effectiveness of betahistine treatment for benign paroxysmal positional vertigo. Hearing, Balance and Communication.

[125] Lee J-YLI-B Kim M-B. Correlation between residual dizziness and modified clinical test of sensory integration and balance in patients with benign paroxysmal positional Vertigo. Res Vestibul Sci. (2021) 23:93–100.

[126] Hui J Lei Q Ji Z Zi D. Betahistine alleviates benign paroxysmal positional vertigo (BPPV) through inducing production of multiple CTRP family members and activating the ERK1/2-AKT/PPARy pathway. Biol Res. (2022) 55:16. doi: 10.1186/s40659-022-00385-3, PMID: 35379352 PMC8981858

