# Corrigendum: Residual dizziness after BPPV management: exploring pathophysiology and treatment beyond canalith repositioning maneuvers

**DOI:** 10.3389/fneur.2024.1461600

**Published:** 2024-07-29

**Authors:** O. Nuri Özgirgin, Herman Kingma, Leonardo Manzari, Michel Lacour

**Affiliations:** ^1^Bayındır Sogutozu Hospital, Ankara, Türkiye; ^2^Faculty of Medicine, Aalborg University, Aalborg, Denmark; ^3^Maastricht University Medical Center, Maastricht, Limburg, Netherlands; ^4^Vestibology Science, MSA ENT Academy Center, Cassino, Lazio, Italy; ^5^Aix-Marseille Université, Neurosciences Department, Marseille, France

**Keywords:** residual dizziness, benign paroxysmal positional vertigo, vestibular compensation, holistic, pathophysiology

In the published article, the reference for 29 was incorrectly written as Yu J, Yu Q, Guan B, Lu Y, Chen C, Yu S. Pseudo-benign paroxysmal positional Vertigo: a retrospective study and case report. *Front Neurol*. (2020) 11:187. 10.3389/fneur.2020.00187

It should be Power L, Murray K, Bullus K, Drummond KJ, Trost N, Szmulewicz DJ. Central conditions mimicking benign paroxysmal positional Vertigo: a case series. *J Neurol Phys Ther*. (2019) 43:186–91. 10.1097/NPT.0000000000000276

In the published article, the reference for 31 was incorrectly written as Power L, Murray K, Bullus K, Drummond KJ, Trost N, Szmulewicz DJ. Central conditions mimicking benign paroxysmal positional Vertigo: a case series. *J Neurol Phys Ther*. (2019) 43:186–91. 10.1097/NPT.0000000000000276

It should be Power L, Murray K, Szmulewicz DJ. Characteristics of assessment and treatment in benign paroxysmal positional vertigo (BPPV). *J Vestib Res*. (2020) 30:55–62. 10.3233/VES-190687

In the published article, the reference for 33 was incorrectly written as Roberts RA, Gans RE, Kastner AH. Differentiation of migrainous positional vertigo (MPV) from horizontal canal benign paroxysmal positional vertigo (HC-BPPV). *Int J Audiol*. (2006) 45:224–6. 10.1080/14992020500429658

It should be Yu J, Yu Q, Guan B, Lu Y, Chen C, Yu S. Pseudo-benign paroxysmal positional Vertigo: a retrospective study and case report. *Front Neurol*. (2020) 11:187. 10.3389/fneur.2020.00187

In the published article, the reference for 116 was incorrectly written as Power L, Murray K, Szmulewicz DJ. Characteristics of assessment and treatment in benign paroxysmal positional vertigo (BPPV). *J Vestib Res*. (2020) 30:55–62. 10.3233/VES-190687

It should be Lee S-H, Kim JS. Benign Paroxysmal Positional Vertigo. *J Clin Neurol*. (2010) 6:51–63.10.3988/jcn.2010.6.2.51

In the published article, there was an error made in correctly attributing four references. A correction has been made to **Diagnosis of BPPV**, paragraphs 1 and 2. This sentence previously stated:

“Suspicion of central positional vertigo may be raised with an absence of latency or fatigability of nystagmus, a lack of marked vertigo, pure upbeat or downbeat nystagmus, or as a lack of responsiveness to CRM (31). Therefore, it is recommended that a diagnosis of BPPV can only be made if the supine roll maneuver Semont or Dix-Hallpike tests elicit nystagmus that is consistent with BPPV; any features of the nystagmus not consistent with BPPV should raise suspicion of central pathology and warrant further investigation (31).

There is increasing evidence about the association between vestibular migraine (VM) and BPPV (29). Despite their similarities, BPPV can be differentiated from VM by the direction of the nystagmus and the duration of the symptoms (32, 33). Although there is generally no positional nystagmus in VM, pseudo-BPPV is a complex mix of positional, atypical positional and non-positional vertigo accompanied by migraine features; the ability to distinguish pseudo-BPPV from other vertigo disease has great clinical significance for treatment (29).”

The corrected sentence appears below:

“Suspicion of central positional vertigo may be raised with an absence of latency or fatigability of nystagmus, a lack of marked vertigo, pure upbeat or downbeat nystagmus, or as a lack of responsiveness to CRM (29). Therefore, it is recommended that a diagnosis of BPPV can only be made if the supine roll maneuver Semont or Dix-Hallpike tests elicit nystagmus that is consistent with BPPV; any features of the nystagmus not consistent with BPPV should raise suspicion of central pathology and warrant further investigation (29).

There is increasing evidence about the association between vestibular migraine (VM) and BPPV (31). Despite their similarities, BPPV can be differentiated from VM by the direction of the nystagmus and the duration of the symptoms (32). Although there is generally no positional nystagmus in VM, pseudo-BPPV is a complex mix of positional, atypical positional and non-positional vertigo accompanied by migraine features; the ability to distinguish pseudo-BPPV from other vertigo disease has great clinical significance for treatment (33).”

The authors apologize for this error and state that this does not change the scientific conclusions of the article in any way. The original article has been updated.

